# Phenotypic spectrum of *RNU4ATAC*-related spliceosomopathies: four novel cases and integrated reevaluation of previously reported patients

**DOI:** 10.1186/s13023-026-04300-x

**Published:** 2026-03-10

**Authors:** Svjetlana Lovric, Ann-Cathrine Berking, Felix C. Ringshausen, Julia Körholz, Joseph Porrmann, Sylvia Hütter, Jan H. Bräsen, Nataliya di Donato, Kai M. Schmidt-Ott, Torsten Witte, Sandra von Hardenberg, Georgios Sogkas

**Affiliations:** 1 Department of Nephrology and Hypertension, Hannover Medical School, Hannover, Germany; 2 Department of Human Genetics, Hannover Medical School, Hannover, Germany; 3 Department of Respiratory Medicine and Infectious Diseases, Hannover Medical School, Hannover, Germany; 4 Biomedical Research in End-Stage and Obstructive Lung Disease Hannover (BREATH), German Center for Lung Research (DZL), Hannover, Germany; 5 European Reference Network on Rare and Complex Respiratory Diseases (ERN-LUNG), Frankfurt, Germany; 6 Department of Pediatrics, Faculty of Medicine, University Hospital Carl Gustav Carus, Technische Universität Dresden, Dresden, Germany; 7 Institute for Clinical Genetics, University Hospital Carl Gustav Carus, Technische Universität Dresden, Dresden, Germany; 8 Nephropathology Unit, Institute of Pathology, Hannover Medical School, Hannover, Germany; 9 Hannover Medical School, Cluster of Excellence RESIST (EXC 2155), Hannover, Germany; 10 Department of Rheumatology and Immunology, Hannover Medical School, Carl-Neuberg-Strasse 1, 30625 Hannover, Germany

**Keywords:** *RNU4ATAC*, Immunodeficiency, Inborn errors of immunity, MOPD1, Taybi-Linder syndrome, Lowry-Wood syndrome

## Abstract

**Background:**

Homozygous or compound heterozygous variants in *RNU4ATAC*, which transcribes a non-coding RNA component of the minor spliceosome, have been associated with a spectrum of disorders, collectively known as *RNU4ATAC*-related spliceosomeopathies. The phenotypic spectrum of *RNU4ATAC*-related disease is characterized by dysmorphic features, growth delay, neurological and skeletal features, whose severity ranges from the microcephalic osteodysplastic primordial dwarfism type 1 (MOPD1) to the milder Roifman syndrome.

**Objectives:**

To characterize the clinical spectrum and evaluate long-term outcomes of *RNU4ATAC*-related diseases.

**Methods:**

We evaluated the phenotypic features of four novel patients with deleterious *RNU4ATAC* variants, diagnosed by means of whole genome sequencing. Same features were evaluated in previously published cases, identified by literature research on PubMed.

**Results:**

We identified four novel cases with deleterious compound heterozygous variants in *RNU4ATAC*, which were not restricted to the 5’ stem-loop, including three adult patients. Reported cases expand the clinical spectrum of *RNU4ATAC*-related disorders, highlighting renal disease, autoimmunity and systemic inflammation as possibly more frequent yet previously under-recognized features. Immunological investigations reveal enhanced HLA-DR and PD-1 expression in T cells from tested patients, suggesting T cell activation and exhaustion. Reevaluation of all previously published cases confirms the strong correlation of *RNU4ATAC* variants located exclusively at the 5’ stem-loop with severe lethal disease falling under MOPD1. Genotypes carrying at least one variant that spares the 5′ stem-loop are associated with a milder phenotype and later onset.

**Conclusion:**

Homozygous or compound heterozygous *RNU4ATAC* variants affecting the 5’ stem-loop region are associated with severe phenotypes and adverse disease courses. In contrast, genotypes sparing the critical 5′ stem-loop region of *RNU4ATAC* can cause a complex phenotype that is not necessarily dominated by dysmorphic features or growth failure, but rather by immunodeficiency and immune dysregulation.

**Supplementary Information:**

The online version contains supplementary material available at 10.1186/s13023-026-04300-x.

## Introduction

*RNU4ATAC* transcribes the small nuclear RNA (snRNA) U4atac, a core component of the minor or U12-dependent spliceosome, which removes minor introns, residing in approximately 750 human genes [1–3]. The 3’ stem loop of U4atac is followed by a single strand region serving as the binding site of Sm proteins, required for the maturation and stability of U4atac [4–6]. The stem I and stem II elements of U4atac pair with U6atac and are separated by the 5’ stem-loop. Although U4atac itself is not catalytically active, it base-pairs U6atac, thereby stabilizing and enabling U6atac to execute its catalytic function. Biallelic variants in *RNU4ATAC* cause heterogenous disorders, including the microcephalic osteodysplastic primordial dwarfism type I (MOPD1; OMIM #210710), also named Taybi-Linder syndrome, the Roifman (RS; OMIM #616651) and the Lowry-Wood syndrome (LWS; OMIM #226960) [7–10]. All three *RNU4ATAC*-related spliceosomopathies – collectively referred to as *RNU4ATAC*-opathies – share a common core phenotype characterized by dysmorphic facial features, growth retardation, and neurological and skeletal involvement of variable severity [11]. In contrast to MOPD1, LWS and RS are associated with a different clinical spectrum, including disease-specific dysmorphic features and survival extending into late childhood or adulthood. Immunodeficiency, retinal anomalies and variable organ-related manifestations have been rather associated with RS and have likewise been reported in LWS.

The majority of *RNU4ATAC* variants associated with MOPD1 are located exclusively at the 5’ stem loop, whereas variants associated with RS are typically compound heterozygous, with one variant at the 5’ stem loop or the Sm protein binding element and the second variant at the stem II region [12]. The higher pathogenic potential of variants located at the 5’ stem loop region, reflected by the profound disease severity in patients with variants located exclusively at the 5’ stem loop, may be explained through the severely impaired binding of U4atac to key protein constituents of the minor spliceosome (i.e. NHP2L1 and PRPF31) [9, 13, 14].

Defective minor intron splicing as a consequence of deleterious biallelic variants in *RNU4ATAC* results in intron retention and elevated alternative splicing, affecting the tissue-specific distribution of mRNA isoforms [15, 16]. The latter can impair the function of minor intron-containing genes and their targets. Olthof et al. detected alternatively spliced minor intron-containing genes in peripheral immune cells from patients with RS or LWS, which suggests the pathogenic effect of aberrations in minor intron splicing in the immune system [15]. Likewise, the neurological, retinal, skeletal and other ciliopathy-consistent manifestations may result from the marked enrichment of cilium-related genes within the subset of genes carrying minor introns [17]. With respect to RS-associated immunodeficiency, altered MAPK1 splicing, leading to impaired BAFF–MAPK1–MAPK3 signaling that normally promotes survival beyond the transitional B-cell phase, has been proposed to account for the aberrant B-cell maturation [9, 16–17].

To date, *RNU4ATAC*-opathies have been commonly reported in young children, most frequently diagnosed with MOPD1. The clinical spectrum and outcome of patients with milder disease remains barely characterized. To this end, we evaluated the phenotypic features of four novel patients with deleterious *RNU4ATAC* variants. Our cases, especially adult ones, highlight immune dysregulation in the form of autoimmunity and systemic inflammation as later-onset feature of *RNU4ATAC*-related spliceosomopathies. Review of previously reported cases suggests the phenotypic continuum of *RNU4ATAC*-opathies with two clearly opposing ends of severity.

## Materials and methods

### Patient cohort and collection of clinical data

This retrospective cohort study included patients, visiting the outpatient clinics of the Hannover Medical School or the Dresden University Hospital. Available clinical and laboratory data recorded until 06/2025. All patients and parents provided written informed consent and the study was approved by the Ethics Committee of the Hannover Medical School (approval No. 11223_BO_K_2024). Clinical data were obtained from patients’ medical files as described previously [18]. Recorded phenotypic features included body measurements (weight, height, head circumference) and features of cranial and facial dysmorphisms. All diagnoses, imaging studies and laboratory findings, including immunological investigations and clinical chemistry results (including C-reactive protein (CRP), liver enzymes, renal function markers and urine investigations) were reviewed retrospectively.

### Genetic analysis

Sample preparation, whole genome sequencing (WGS) analysis and the assessment of rare single nucleotide variants (SNV) were performed as described previously [19]. Briefly, genomic DNA was extracted from peripheral whole blood samples collected in tubes with ethylenediaminetetraacetic acid (EDTA) and sequenced on Illumina NovaSeq 6000 using the Lotus DNA Library Prep Kit (IDT, Leuven, Belgium) or the DNA PCR-Free Prep Kit (Illumina, United States). 150-bp paired-end reads were generated, aligned to the human reference genome (UCSC Genome Browser build GRCh38/hg38) and analyzed with the next-generation sequencing (NGS) data analysis pipeline (megSAP v.0.2-8-ga9d80c2 or TruSight^™^ Software Suite (illumine)). SNVs were filtered for genes associated with inborn error of immunity (IEI) and classified following the American College of Medical Genetics (ACMG) Standards and Guidelines [20]. Follow-up of SNVs was performed using databases Exome Aggregation Consortium19 (ExAC), the 1000 Genomes Project and the genome Aggregation Database (gnomAD) for filtering for rare variants (≤ 1% minor allele frequency, MAF). The Leiden Open Variation Database (LOVD), ClinVar and prediction tools including REVEL, phyloP, SIFT, PolyPhen2, FATHMM, and CADD were used for evaluation of pathogenicity.

### Systematic review of published cases with *RNU4ATAC*-related spliceosomopathies

Cases reported until 06/2025 were reviewed using PubMed search term: “Roifman syndrome” OR “MOPD1” OR “Lowry-Wood syndrome”, following the Preferred Reporting Items for Systematic Reviews and Meta-Analyses (PRISMA) guidelines (Suppl. Figure 1) [21]. Cases without documented biallelic variants as well as embryonic and fetal cases were not considered. All available genotypic, phenotypic and immunological data were abstracted and summarized together with the data from the here presented cases. Evaluated data included patient demographic data, the diagnosis of RS, LWS or MOPD1, reported intrauterine growth retardation (IUGR), small for gestational age (SGA), postnatal growth delay, dysmorphic features (facial dysmorphism, dysmorphic digits (including brachydactyly, syndactyly, clinodactyly), dysmorphic limbs), cardiac abnormalities, infectious manifestations and results of immunological investigations (immunoglobulin levels, vaccine responses, B cell and T cell counts). Patients diagnosed with MOPD1 were considered to present with cranial dysmorphism in the form of microcephaly, along with prenatal and postnatal growth retardation, unless otherwise specified in the original case reports. Reported hypogammaglobulinemia, dysgammaglobulinemia, failed vaccine response or reported severe or frequent infections were collectively evaluated as the presence of “clinical or immunological evidence of immunodeficiency”. Reported joint contractures, dislocations, scoliosis, kyphosis and epiphyseal or spondyloepiphyseal dysplasia were also collectively evaluated as “skeletal abnormalities”. Patients reported with vermian hypoplasia were documented to display cerebellar hypoplasia. Reported intracranial cysts, vermian hypoplasia or dysplasia, cerebellar hypoplasia, gyration abnormalities or dysgenesis of the corpus callosum were collectively evaluated as “brain malformation”. Patients diagnosed with atopic dermatitis were documented with eczema. In case of reported ichthyosis, we also documented dry skin. Chilblain, eczema or dry skin, were also collectively evaluated as “skin abnormalities”.

### Statistical analysis

For statistical calculation we used GraphPad prism 9 (GraphPad, La Jolla, USA). Descriptive statistics are reported as median and interquartile range (IQR) in case of continuous variables and as counts and percentages for dichotomous variables. Categorical variables were compared by the Yate’ s continuity corrected chi-squared test, which was employed to compare patients with 5’ stem loop variants exclusively with the rest of documented patients. Differences in lymphocyte counts between examined patients and matched healthy blood donors were evaluated with the Mann-Whitney test. Probabilities of survival were compared with the Gehan-Breslow-Wilcoxon test.

## Results

### Case reports

**Fig. 1:**
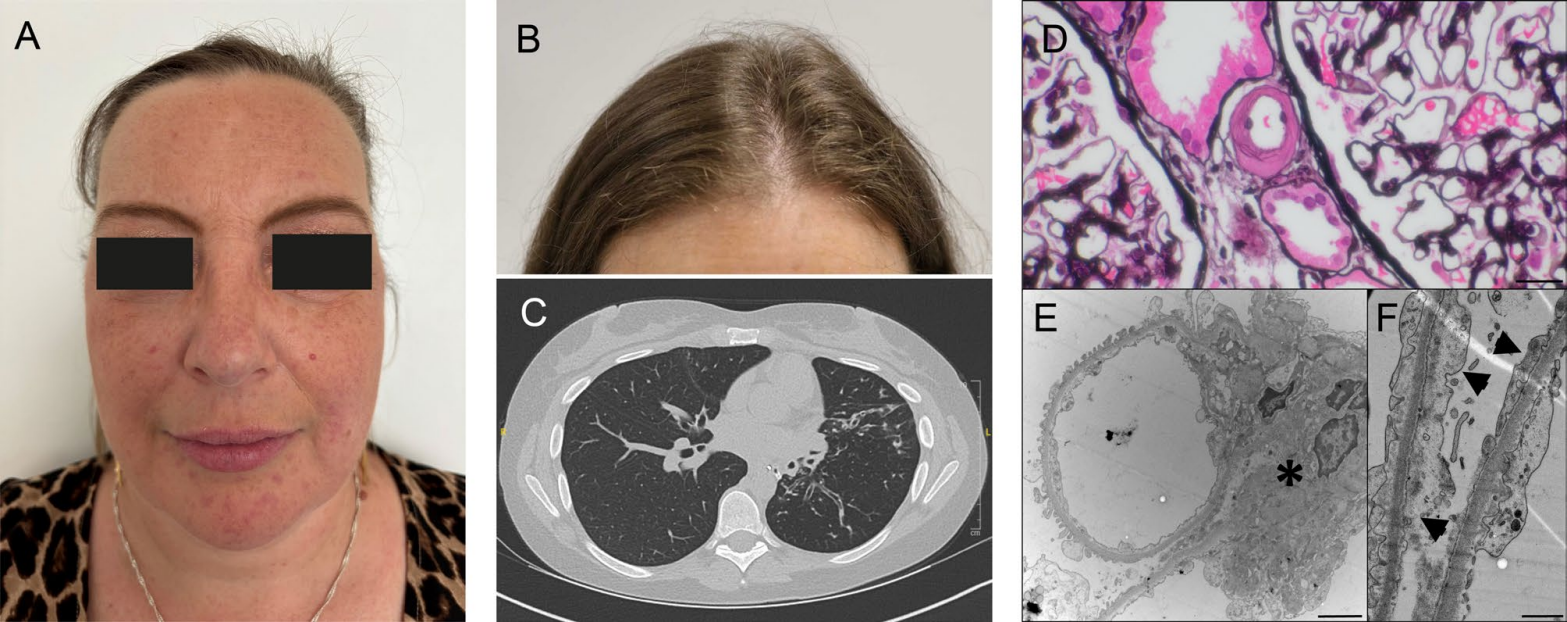
Features of patients with RNU4ATAC-related spliceosomopathy. P1 with prominent nose, high nasal root and broad nasal bridge (**A**). Sparse scalp hair at frontal hairline in case of P2 (**B**). Computed tomography scan from P2 showing cylindrical bronchiectasis of left upper lobe (marked with “*”, **C**). Kidney biopsy from P3 (right) revealing severe transmural and cuff-shaped hyalinosis of small preglomerular vascular branches with loss of smooth muscle cells (marked with an arrow, **D**) (Jones’ methenamine combined with hematoxylin and eosin stain; bar represents 20 μm). Electron microscopy showing small segmental glomerular sclerosis (marked with “*”, **E**) and 30% loss of podocyte foot processes (marked with arrowheads, **F**) (electron microscopy after contrasting with osmium tetroxide and uranyl acetate in **E** and **F**; bars represent 2,500 nm in **E**, and 1,000 nm in **F**)

**P1** is a 42-year-old female with a history of recurrent sinusitis and otitis media since approximately the age of 30 years, leading to frequent antibiotic treatment (Fig. 1; Table 1). Her medical history included congenital asplenia, atopic dermatitis presenting already in early childhood and Hashimoto’s thyroiditis, leading to treatment with levothyroxine. Shortly before her first presentation at our immunology outpatient clinic, increased serum creatinine and non-nephrotic proteinuria associating with elevated perinuclear antineutrophil cytoplasmic antibodies (ANCA) against myeloperoxidase (MPO), led to treatment with high-dose prednisolone. Prednisolone treatment improved serum creatine and reduced proteinuria. Suspecting an ANCA-positive vasculitis, a kidney biopsy was recommended; however, the patient declined the procedure. A computed tomography scan of the chest revealed no lesions suggestive of ANCA-positive vasculitis, interstitial lung disease or bronchiectasis. Recurrent upper respiratory tract infection led to immunological investigations prior to the introduction of prednisolone treatment, revealing reduced IgA and IgG4 levels. During high-dose steroid treatment, total IgG levels were also transiently low and recovered after tapering of prednisolone. Given diagnosed dysgammaglobulinemia and recurrent infections, we introduced a prophylactic treatment with azithromycin, which reduced the frequency of infections. Genetic testing by means of whole genome sequencing identified compound heterozygous variants in *RNU4ATAC* gene (Table 1) and led to the diagnosis of an *RNU4ATAC*-related spliceosomopathy.

**Table 1:** Phenotypic features of four novel patients with biallelic deleterious variants in *RNU4ATAC*

Pat. ID	RNU4ATAC variants	Sex	Age*	Growth failure	Cranial/Facial dysmorphism	Other Syndromic features	Skeletal features	Infections	Neurological features	Ophalomological features	Endocrinological features	Integumentary features	Kidney disease	Other features
1	n. 8 C > T; n. 51G > A	F	43.2	no	prominent nose, high nasal root, broad nasal bridge, long palpebral fissures	broad thumbs, thick fingers	scoliosis, osteoarthritis of the spine	recurrent URTI, recurrent bronchitis	no	myopia (OD -1.25 D/ OS -8.25 D)	Hashimoto’s thyroiditis leading to hypothyroidism	atopic dermatitis, rosacea, sparse scalp hair at frontal hairline	ANCA associated vasculitis-like syndromes with chronic kidney disease, CKD G3aA3, creatinine 105 µmol/l (eGFR 54 ml/min/1.73m^2^), S-cystatin C 1.29 mg/l, (cystatin C clearance 55 ml/min/1.73 m^2^); urine findings in spot urine: UPCR ratio 406 mg/g creatinine; UACR ratio 275 mg/g creatinine; urine sediment with erythrocyturia & leukocyturia	congenital asplenia, elevated liver enzymes (both ALT & gGT)
2	n. 8 C > T; n. 37G > A	F	32.3	no	microcephaly, high nasal root, broad nasal bridge, deep set eyes, long palpebral fissures, thin upper lip	deep set eyes	scoliosis	recurrrent URTI & LRTI (including otitis media, bronchitis and pneumonia), bronchiectasis *Helicobacter pylori*–related gastritis, cylindrical bronchiectasis in the right upper lobe, middle lobe & lingula, status post resection of the left upper lobe of the lungs, recurrent herpes labialis, common warts at fingers, herpes zoster	no	myopia, astigmatism	no	sparse scalp hair at frontal hairline, laterally thinned eyebrows	CKD G2A2 creatinine 89 µmol/l (eGFR 85 ml/min/1.73m^2^), S-cystatin C 1,16 mg/l, (cystatin C clearance 66 ml/min/1.73 m^2^), urine findings in spot urine: UPCR ratio 386 mg/g creatinine, UACR ratio 47 mg/g creatinine; urine sediment unremarkable	no
3	n. 13 C > T; n. 117 A > G	F	37.6	SGA (likely also IUGR), dwarfism	sloping forehead, high nasal root, broad nasal bridge, dysplastic ears, long midface	bilateral 5th finger clinodactyly, brachydactyly	spondyloepimetaphyseal dysplasia, reduced bone age, genua valga, short metacarpals	recurrrent URTI & LRTI (including pneumonia, bronchitis & sinusitis), bronchiectasis at middle lobe, Salmonella enteritis, cutaneous abscesses, severe COVID-19 (leading to hospitalization), vulvar cancer (pT1a pN0 L0 V0 Pn0 R0), associated with HPV 44, 45 and 54 (treated with vulvectomy), thrush (single episode)	delayed motor milestones	retinitis pigmentosa, intermittent divergent strabismus	IGF1 deficiency, thyreoiditis	dry skin, transient ichthyosis, lichen ruber, sparse scalp hair, laterally thinned eyebrows	nephrotic range proteinuria, CKD G4A2, creatinine 213 µmol/l (eGFR 25 ml/min/1.73m^2^), S-cystatin C 3.34 mg/l, (cystatin C clearance 16 ml/min/1.73m^2^). progressing to CKD; kidney biopsy: Severe transmural and cuff-shaped hyalinosis of small preglomerular vascular branches with loss of smooth muscle cells. Electron microscopy reveals a small segmental glomerular sclerosis and 30% loss of podocyte foot processes. urine findings in spot urine: UPCR ratio 227 mg/g creatinine; UACR ratio 52 mg/g creatinine; urine sediment with leukocyturia	gout, hyperuricemia, cholestatic hepatopathy, splenomegaly
4	n. 13 C > T; n. 51G > A	M	8.0	IUGR and postnatal growth retardation	microcephaly, dolichocephaly, long philtrum, micrognathia, upslanting palpebral fissures, large fleshy & upturned nose with hypoplastic alae nasi, low-set & posteriorly rotated ears	bilateral 5th finger clinodactyly, brachydactyly, bilateral transverse palmar creases	genua valga	recurrent mild URTI (rhinitis, cough, otitis media), chronic otitis media, conjunctivitis	unilateral (left) polymicrogyria, right hemiparesis, muscular hypotonia at neonatal age, delayed motor milestones, intellectual disability, mental retardation	no	no	dry and irritable skin, atopic dermatitis, long prominent eyelashes	no	patent foramen ovale, conductive hearing loss, micropenis, cryptorchidism leading to orchidopexy and funicolysis

ALT, Alanine transaminase; ANCA, anti-neutrophil cytoplasmic antibody; CKD, chronic kidney disease; COVID-19, coronavirus disease 2019; D, diopters; F, female; gGT, gamma-glutamyltransferase; HPV, human papillomavirus; IGF1, insulin-like growth factor 1; IUGR, intrauterine growth retardation; LRTI, lower respiratory tract infections; M, male; S, serum; UPCR, urine protein creatinine ratio; SGA, small for gestational age; UACR, urine albumin creatinine ratio; URTI, upper respiratory tract infections

*at reporting

**P2** is a 33-year-old female with a history of recurrent respiratory tract infections, including bronchitis and otitis media since the age of 1–2 years (Fig. 1; Table 1). She first developed pneumonia at the age of 12 years. Since then, she suffered from recurrent pneumonias and at the age of 18, she was diagnosed with bilateral cylindrical bronchiectasis (Fig. 1), leading to resection of the upper left lobe of the lung. At the age of 17, she was diagnosed with herpes zoster for the first time. Furthermore, she had a history of recurrent episodes of herpes labialis and refractory confluent common warts affecting the digits at her extremities. Immunological investigations revealed low lgG2 and IgG3 antibodies and led to the diagnosis of selective subclass deficiency and the introduction of immunoglobulin replacement therapy, which the patient tolerated well in its subcutaneous form. Immunoglobulin replacement therapy reduced infection rate. Similar to P1, this patient displayed mildly increased serum creatinine and non-nephrotic proteinuria.

**P3** is a 37-year-old female who was born with low birth weight, corresponding to the 6th percentile (Table 1). At the age of 10 weeks, she was diagnosed with a bilateral pneumonia. During early childhood she experienced recurrent episodes of pyelonephritis, attributed to grade 2 vesicoureteral reflux. At the age of 7 years, recurrent episodes of pneumonia and bronchitis led to immunological investigations and the diagnosis of humoral immunodeficiency with low IgM and IgG4 as well as markedly reduced isoagglutinin levels. Consequently, an immunoglobulin replacement treatment was introduced, leading to reduced infection frequency. Ophthalmologic assessment at 3.5 years raised suspicion of cone-rod dystrophy. Worsening visual acuity led to follow-up ophthalmologic investigations at the age of 12 years, whose findings were consistent with a retinitis pigmentosa. Dwarfism, dysmorphic features, including brachydactyly and clinodactyly as well as features of skeletal dysplasia were consistent with a syndromic disorder. At 19 years of age, she developed mild proteinuria, which progressed over the next five years to nephrotic-range proteinuria. This prompted a kidney biopsy, revealing severe segmental hyalinosis of the smaller preglomerular vascular branches, with partly transmural and cuff-shaped lesions and segmentally marked loss of smooth muscle cells (Fig. 1). Electron microscopy demonstrated a small segmental sclerosis and 30% loss of podocyte foot processes (Fig. 1D and E). These findings excluded an immune-mediated glomerulonephritis, did not fulfil the criteria of podocytopathy and did not correspond to any known glomerulopathy. An ultrasound of the kidney at the age 32, showed increased echogenicity. The right kidney was smaller than the left kidney but no vesicoureteral reflux, cystic or dysplastic kidneys were detectable. The patient was treated with an angiotensin-converting enzyme inhibitor, resulting in a mild reduction in proteinuria. Her nephropathy subsequently progressed slowly to chronic kidney disease. The combination of immunodeficiency, nephropathy, and skeletal abnormalities suggested immuno-osseous dysplasia, which could not be confirmed by whole exome sequencing (WES). Consistent with three other cases reported here, the diagnosis of *RNU4ATAC*-related spliceosomopathy was ultimately established using whole genome sequencing (WGS) (Table 1).

Finally, **P4** was an 8-year-old male patient, born hypotrophic at 35 weeks of gestation. At birth, he presented with primary microcephaly and generalized hypotonia. Over the course of early infancy, he developed progressive failure to thrive and short stature. A combined motor and speech developmental delay became evident from 2 to 3 months of age, leading to placement in a special-needs school at the age of 7 years. Neurological examination revealed decreased spontaneous movement of the right extremities with spastic paresis, which showed slight improvement over time. Since his infancy, the patient experienced recurrent respiratory tract infections, averaging 1–2 episodes per month, along with repeated purulent otitis media. At the age of 5, he underwent otolaryngologic surgery, including paracentesis and tympanostomy tube insertion. Remarkably, no hospitalizations for severe infections have occurred. All recommended vaccinations were administered without complications. Following vaccination, the patient exhibited protective serum antibody levels to tetanus and measles. Neuroimaging revealed temporo-occipital polymicrogyria, white matter thinning and consequent enlargement of the left lateral ventricle. Electroencephalogram recordings revealed intermittent epileptiform discharges over the left cerebral hemisphere and the right frontal region, without clinical seizure activity. Echocardiography and renal ultrasonography were unremarkable and serum renal function parameters remained within the normal range so far. The combination of neurodevelopmental delay, failure to thrive and short stature led to genetic testing by means of WGS at the age of six years, revealing two pathogenic *RNU4ATAC* variants leading to the diagnosis of an *RNU4ATAC*-related spliceosomopathy. Follow-up neurological and immunological counselling was established. This patient did not receive any disease-related long-term treatments so far.

### Summary of clinical findings and laboratory results

Considering the current clinical perception of *RNU4ATAC*-related spliceosomopathies, patients P3 and P4, were displaying obvious dysmorphic features and growth abnormalities, i.e. dwarfism and history of IUGR that represent rather typical features of TALS. In contrast, in case of the other two patients (P1 and P2), genetic diagnosis was unexpected given their very mild rather infection-immunodeficiency-dominated phenotype and the lack of apparent dysmorphic features. Furthermore, both those patients demonstrated no evidence of cognitive impairment and maintained normal social and professional functioning. All three adult patients (P1-P3) presented with chronic kidney disease (CKD) associating with proteinuria (Table 1). The presence of ANCA-MPO autoantibodies in P1, along with the improvement in creatinine levels and urine parameters under steroid treatment indicated autoimmunity as a likely cause of renal disease. In P3, however, the absence of hemodynamic, drug-related or other cause of a podocytopathy may suggest a directly *RNU4ATAC*-related etiology of CKD. Further, in all three patients with kidney disease, WGS excluded a monogenic CKD etiology [22] other than the pathogenic *RNUA4ATAC* variants. Clinical findings from all four cases are summarized in Table 1.

Reassessment of laboratory findings, revealed persistently elevated levels of acute phase reactants, including C-reactive protein (CRP). Elevated CRP levels were as well documented also during infection-free intervals in all four patients suggesting chronic systemic inflammation. Noteworthy, among the documented laboratory findings, P1 and P3 showed elevation of liver enzymes, especially of gamma-glutamyl transferase (gGT). The etiology of elevated liver enzymes remained unclear despite standard work-up for viral hepatitis and autoimmune hepatopathies.

### Immunological investigations

**Fig. 2:**
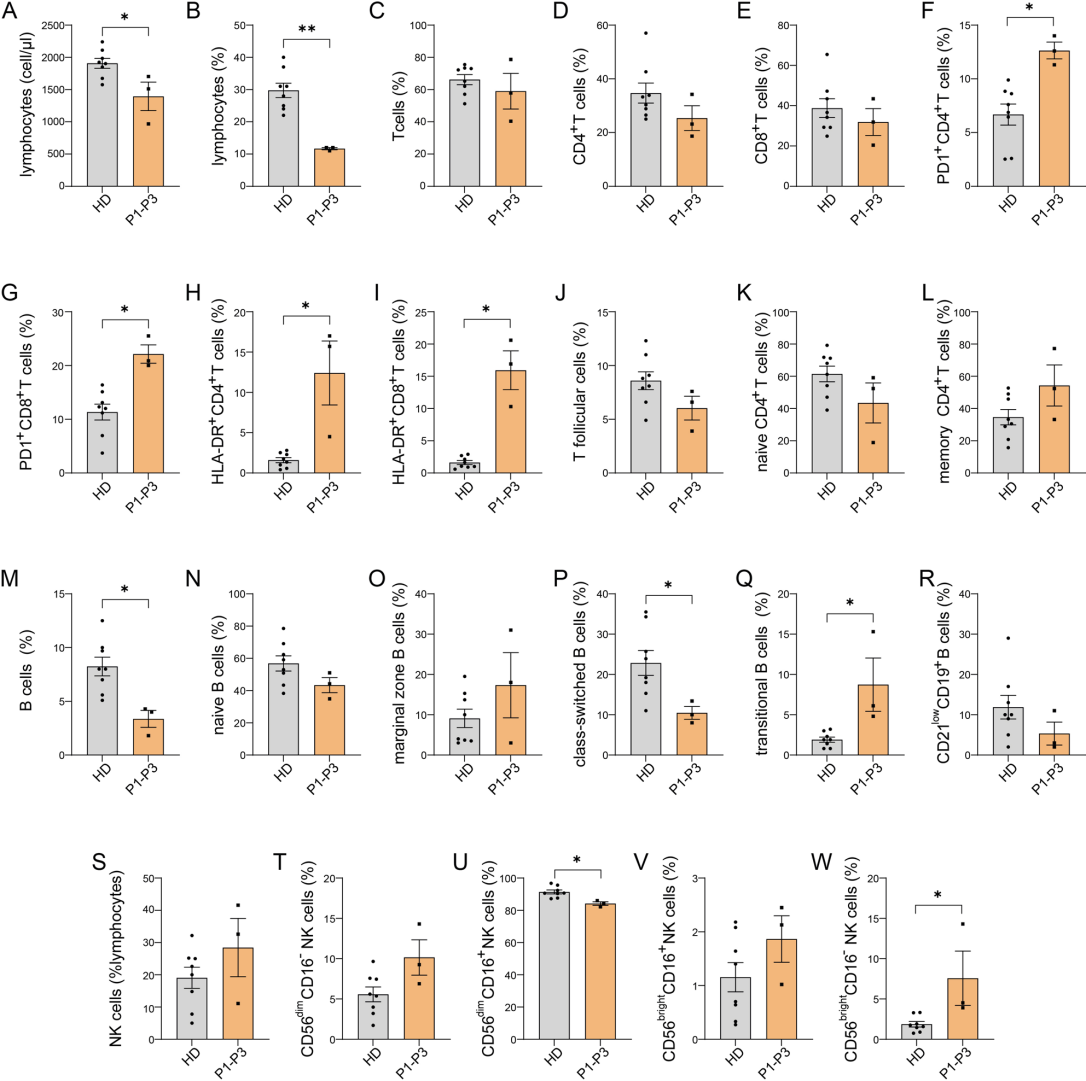
Immunophenotyping of peripheral lymphocytes from three patients with *RNU4ATAC*-related spliceosomopathy. Lymphocyte counts (**A** & **B**), T cell (**C-L**), B cell (**M-R**) and NK cell subset counts (**S-W**). Graphs summarize findings from different healthy blood donors (HD) or patients with *RNU4ATAC*-related spliceosomopathy and indicate the mean ± SEM (differences in lymphocyte counts were evaluated with the Mann-Whitney test; *P < 0.05; **P < 0.01)

Immunological investigations in the pediatric patient (P4) revealed a normal distribution of T-cell subsets. At the age of 6 years, B-cell analysis showed a mild expansion of transitional B cells and CD21^low^ B cells, along with a slightly reduced class-switched IgG^+^ memory B-cell compartment, consistent with a mild B-cell maturation defect. In the adult patients (P1 to P3), immunophenotyping of peripheral lymphocytes was performed uniformly in the same laboratory, following the identical protocol also for parallel-tested age-matched healthy blood donors (HD), who served as the reference group in the absence of established age-related values (Fig. 2). In the context of lymphopenia (Fig. 2A and B), T cells, particularly both CD4^+^ and CD8^+^ T cells displayed higher levels of programmed death-1 (PD-1) and HLA-DR. These findings are indicative of immune exhaustion and T cell activation, respectively (Fig. 2C and L) [23]. The percentages of the rest of studied T cell subsets, including T follicular helper cells, naive and memory CD4^+^ T cells, exhibited no significant differences between the patient and HD groups. Considering the persistently elevated acute phase reactants, increased expression of HLA-DR and PD-1 may be the consequence of active systemic inflammation. With respect to B cells, patients with *RNU4ATAC*-related disease exhibited reduced total counts (Fig. 2M and R). Compared to HD, studied patients showed a decrease in the percentage of class-switched memory cells, while transitional B cells were elevated. The latter observation would be consistent with previous reports evaluating B cell subsets in patients with RS and suggests an aberration in the post-transitional B cell differentiation [13, 24]. In case of NK cells subsets, similar to a previous report [9], we observed a skewing towards CD56^bright^ cells with significant elevation of the CD56^bright^CD16^−^ subset (Fig. 2S and W). Given the reduced cytotoxic potential of this NK cell subset, their elevated counts could be relevant for the susceptibility of studied patients to viral infections. On the other hand, within NK cells, the CD56^bright^CD16^−^ subset has a rather immunoregulatory function [25] and their elevation in *RNU4ATAC*-related disease could play a compensatory role in the context of the above-mentioned systemic inflammation.

### Systematic review of published cases with *RNU4ATAC*-related spliceosomopathies

**Fig. 3:**
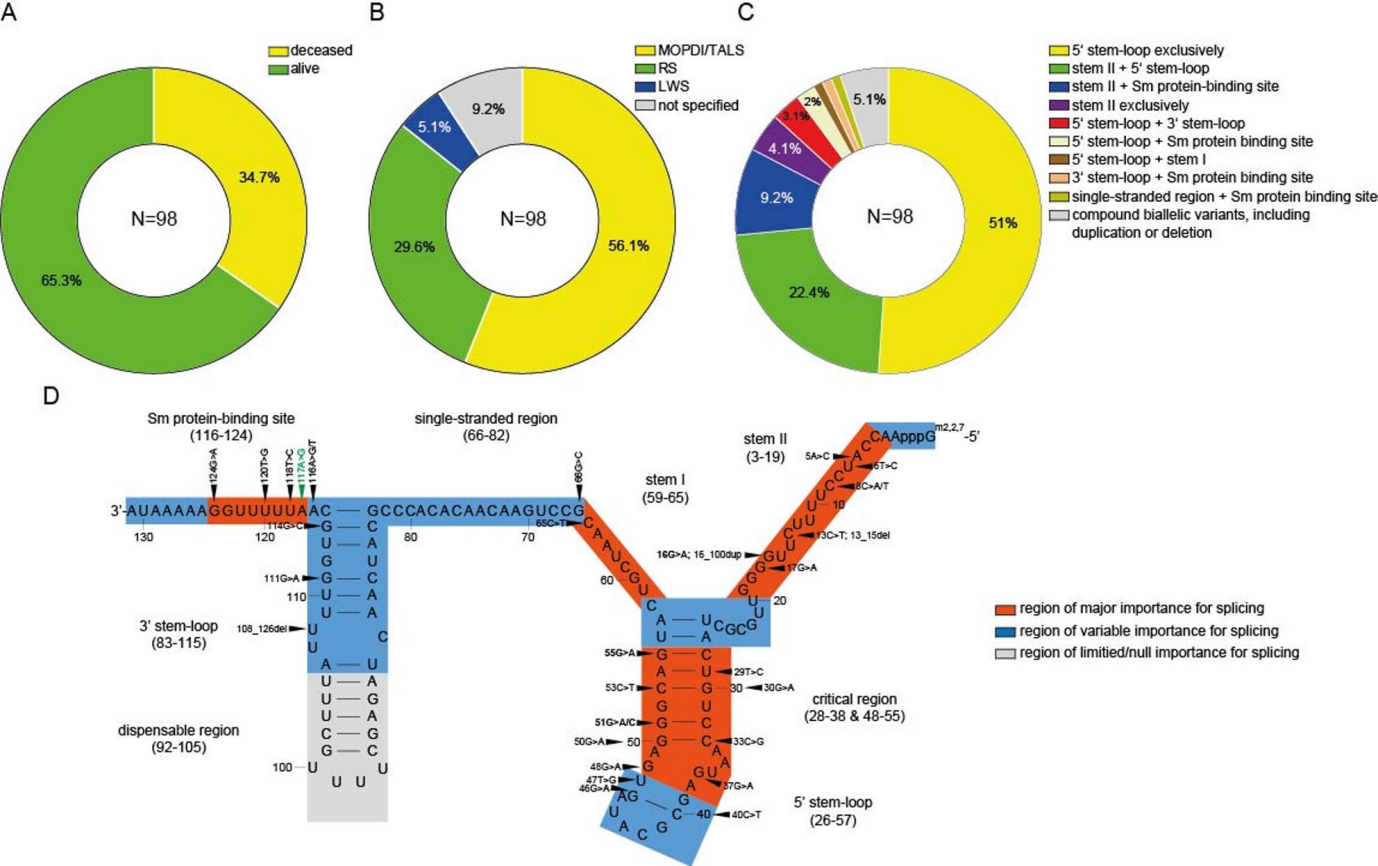
Overview of characteristics and *RNU4ATAC* variants in 98 individuals with *RNU4ATAC*-opathies. Vital status (**A**), clinical diagnosis (**B**) as well as localization of deleterious *RNU4ATAC* variants (**C**). Further, schematic representation of U4atac and summary of reported variants across its regions. (adapted from references 9 & 38, U4atac region definition was based on references 4 & 6) (**D**). The here reported novel variant, identified in P3 is highlighted in green. (LWS, Lowry-Wood syndrome; MOPD1, microcephalic osteodysplastic primordial dwarfism type 1; N, total number; RS, Roifman syndrome; TALS, Taybi-Linder syndrome)

A total of 98 patients with *RNU4ATAC*-opathies were analyzed, including 94 previously published cases that were re-evaluated (Suppl. Table 1) [7–10, 13, 16–17, 26–46] and the four novel cases described above. A fatal outcome was reported in 34 patients (34.7%) (Fig. 3A). The cause of death was specified in only 13 cases and was commonly infection-related, including gastroenteritis (5/13), respiratory tract infection (2/13) and encephalitis (2/13). The majority of reported patients were pediatric cases (Table 2). Only 16/98 (16.3%) were adults, with P1 (43.2 years) being the oldest reported case. The most frequently reported features included growth disorders, microcephaly, craniofacial and digital dysmorphism, brain malformations, skeletal and cutaneous abnormalities, each observed in more than half of the previously described patients (Table 2). More than half of the patients were diagnosed with MOPD1/TALS (55/98, 56.1%), 29/98 (29.6%) with RS and only 5/98 (5.1%) with LWS, while in nine cases (9/98, 9.2%), no clinical diagnosis was reported (Fig. 3B). Identified *RNU4ATAC* variants spanned across all regions of U4atac, except for the distal part of the 3’ stem-loop (Fig. 3C and D). 81/98 patients (82.7%) had at least one *RNU4ATAC* variant in the 5’ stem-loop, while 50/98 (51%) harbored two variants located in the 5’ stem-loop, either homozygous (46/50) or compound heterozygous (4/50). The second most prevalent subgroup of patients with respect to genotype, displayed compound heterozygous variants, with one variant at the 5′ stem-loop and the second at the stem II (22/98, 22.4%).

**Table 2:** Characteristics of 98 patients with *RNU4ATAC*-related spliceosomopathies

Reported as deceased, no. (%)	34 (34.7)
Male sex, no. (%)	44 (44.9)
Median age, years (IQR)	4 (1.15–15.5)
Clinical and/or immunological evidence of immunodeficiency, no. (%)	44 (44.9)
Hypo-/dysgammaglobulinemia, no. (%)	28 (28.6)
Low B cells, no. (%)	22 (22.4)
Severe/recurrent infections, no. (%)	39 (39.8)
URTI, no. (%)	24 (24.5)
LRTI, no. (%)	22 (22.4)
CNS infections, no. (%)	5 (5.1)
GI infections, no. (%)	3 (3.1)
Prenatal growth delay, no. (%)	87 (88.8)
Postnatal growth delay, no. (%)	73 (74.5)
Cranial dysmorphism, no. (%)	82 (83.7)
Microcephaly, no. (%)	80 (81.6)
Trigonocephaly, no. (%)	2 (2)
Facial dysmorphism, no. (%)	68 (69.4)
Limb dysmorphism, no. (%)	22 (22.4)
Digit dysmorphism, no. (%)	50 (51)
Brachydactyly, no. (%)	36 (36.7)
Clinodactyly, no. (%)	16 (16.3)
Syndactyly, no. (%)	5 (5.1)
Retinal dystrophy, no. (%)	24 (24.5)
Strabismus, no. (%)	6 (6.1)
Cataract, no. (%)	4 (4.1)
Optic nerve atrophy, no. (%)	2 (2)
Brain malformation, no. (%)	53 (54.1)
Gyration abnormalities, no. (%)	22 (22.4)
Dysgenesis of corpus callosum, no. (%)	34 (34.7)
Cerebellar hypoplasia, no. (%)	16 (16.3)
Vermian hypoplasia, no. (%)	9 (9.2)
Intracranial cysts, no. (%)	11 (11.2)
Sensineuronal hearing loss, no. (%)	7 (7.1)
Skeletal abnormality/ies, no. (%)	81 (82.7)
Epiphyseal-/spondyloepiphyseal dysplasia, no. (%)	32 (32.7)
Joint contracture/dislocation, no. (%)	20 (20.4)
Scoliosis/kyphosis, no. (%)	11 (11.2)
Agenesis of 12th ribs, no. (%)	5 (5.1)
Skin abnormalities, no. (%)	50 (51)
Dry skin, no. (%)	30 (30.6)
Eczema, no. (%)	11 (11.2)
Chilblain-like, no. (%)	3 (3.1)
Hypoplastic/dysplastic nails, no. (%)	8 (8.2)
Cardiac septal defect, no. (%)	19 (19.4)
Cardiomyopathy, no (%)	1 (1)
Endocrinological features, no. (%)	17 (17.3)
Microdontia or enamel defects, no. (%)	11 (11.2)
Autoimmune/autoinflammatory features, no. (%)	7 (7.1)
Hepato-/splenomegaly, no. (%)	6 (6.1)
Hepatopathy, no. (%)	5 (5.1)
Renal disease, no. (%)	5 (5.1)
Asthma, no. (%)	5 (5.1)
Cryptorchidism, no (%)*	8 (18.2)
Micropenis, no (%)*	6 (13.6)

GI, gastrointestinal; CNS, central nervous system; IQR, interquartile Range; LRTI, lower respiratory tract infections; no., number; URTI, upper respiratory tract infections

*Values represent percentages relative to the male patient subgroup

**Fig. 4:**
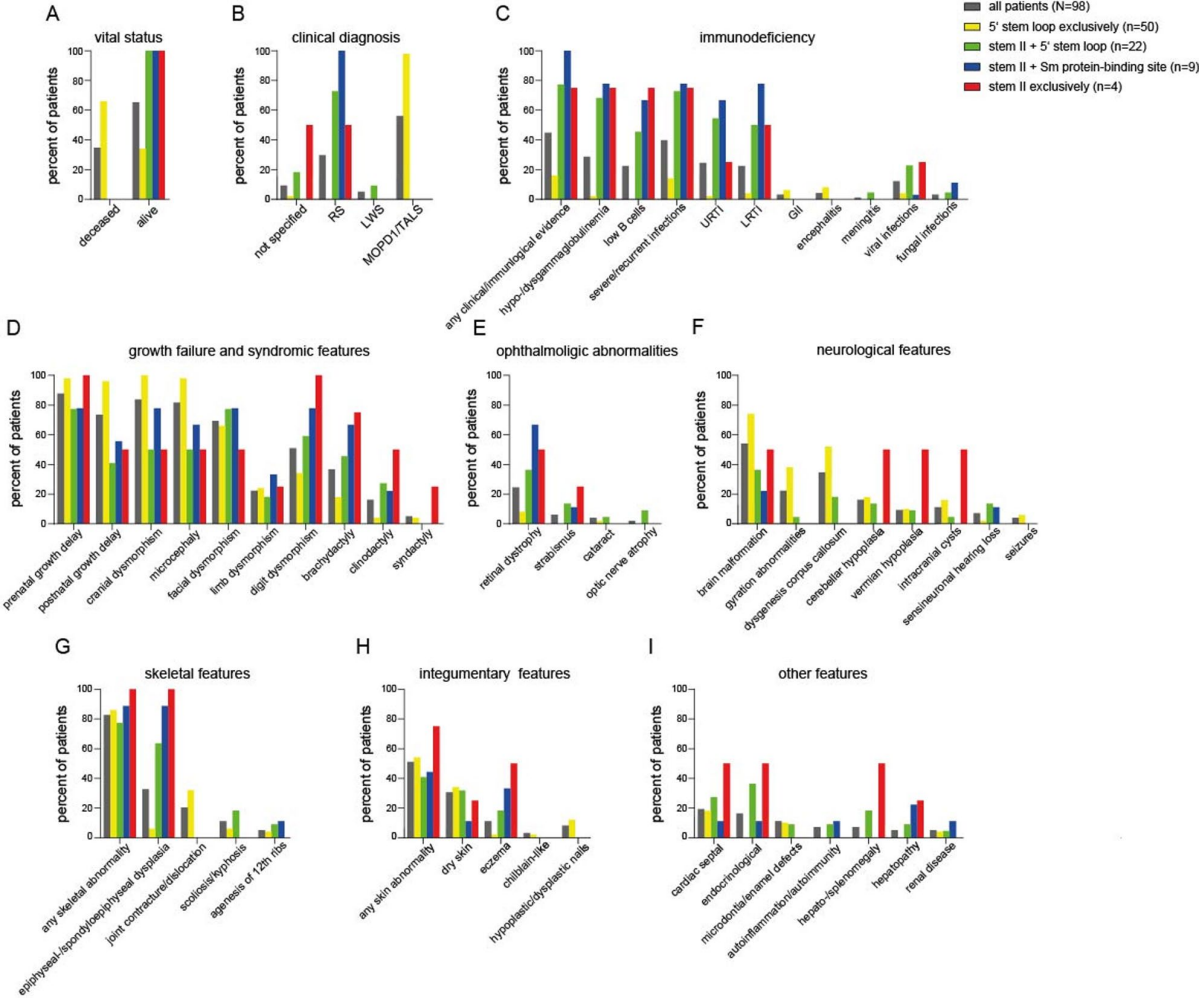
Correlation of genotype with clinical features in 98 patients with RNU4ATAC-related spliceosomopathy. Each panel shows the prevalence of documented manifestations, including vital status (**A**), clinical diagnosis (**B**), immunodeficiency (**C**), growth failure and syndromic features (**D**), ophthalmologic abnormalities (**E**), neurological findings (**F**), skeletal abnormalities (**G**), integumentary manifestations (**H**) and other features (**I**), across genotype-defined patient subgroups. (GII, gastrointestinal infections; LRTI, lower respiratory tract infections; LWS, Lowry-Wood syndrome; MOPD1, microcephalic osteodysplastic primordial dwarfism type 1; n, total number; RS, Roifman syndrome; TALS, Taybi-Linder syndrome; URTI, upper respiratory tract infections)

**Fig. 5:**
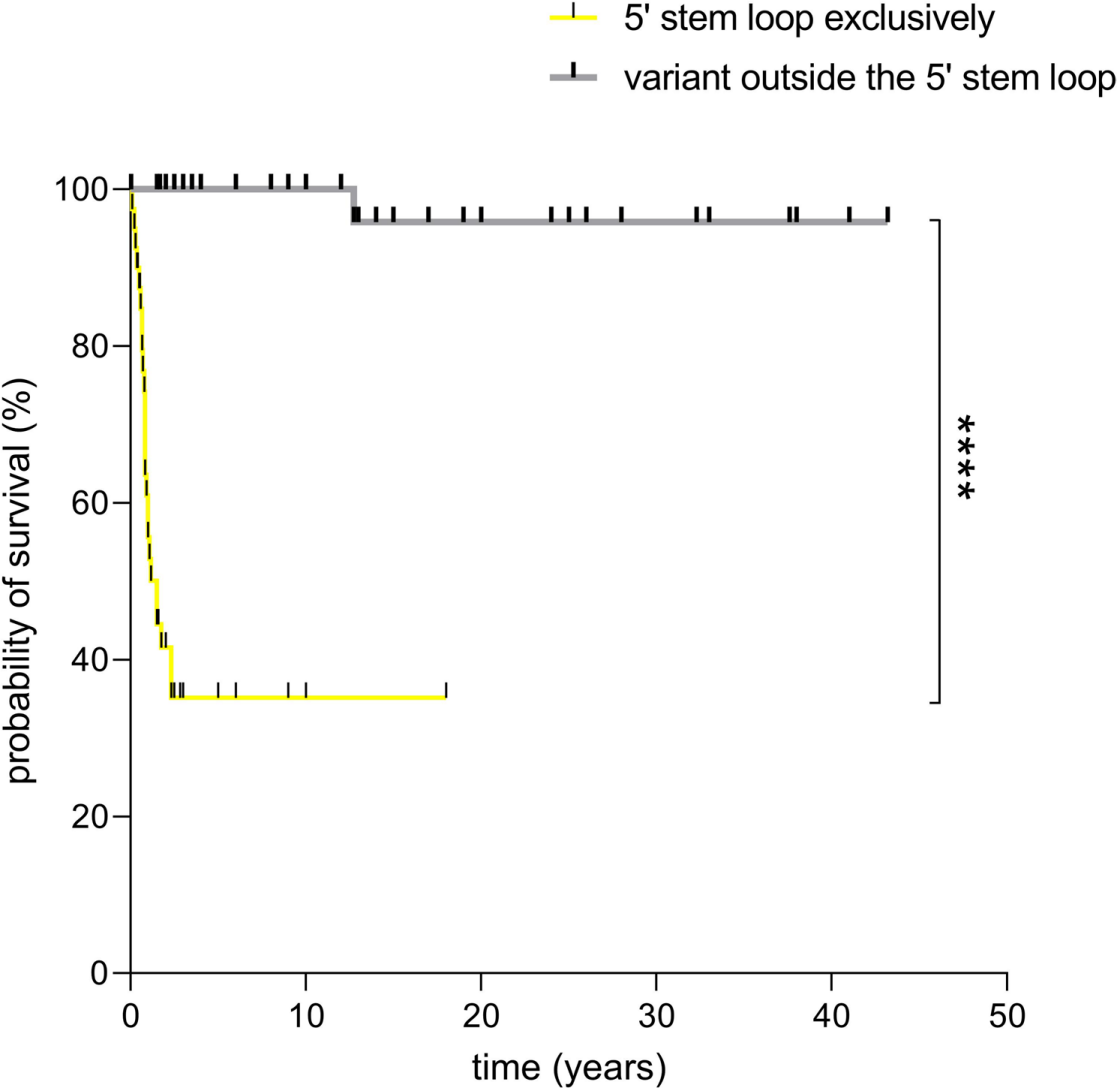
Kaplan-Meier survival curves comparing patients with *RNU4ATAC* variants located exclusively within the 5′ stem-loop versus those with at least one variant outside this region (probabilities of survival were compared with the Gehan-Breslow-Wilcoxon test; ****P < 0.0001)

Except for six cases, MOPD1 was diagnosed in patients carrying variants exclusively within the 5′ stem-loop (Table 3, Fig. 4). Of these six patients, two harbored a variant located at the 5’ stem-loop, while the second variant was a structural-disrupting insertion or deletion that would affect the 5’ stem-loop (suppl. Table 1; n.40 C > T; n.16_100dup). Furthermore, three of these six patients, harbored the n.111G > A variant with either a 5’ stem-loop-located variant (n.30G > A; n.111G > A) or a deletion (n.108_126del; n.111G > A). The strong association of MOPD1 and lethal outcomes (Fig. 5) with variants located in the biologically critical 5′ stem-loop region suggests that more severe *RNU4ATAC*-related phenotypes result from a more profound impairment of U4atac function. Additionally, variants that are exclusively located within the 5’ stem-loop are strongly associated with early childhood mortality, craniofacial dysmorphism and brain malformations, i.e. typical clinical features of MOPD1 (Table 3).

**Table 3:** Frequency of reported clinical features in patients with *RNU4ATAC* variants exclusively inside versus also outside the 5’ stem-loop

	Variants at 5’ stem-loop exclusively (*N* = 50)	Variant outside the 5’ stem-loop (*N* = 48)	OR	95% CI	*p* value^1^ (summary)
Reported as deceased, no. (%)	33 (66)	1 (2.08)	91.24	13.84–949.1	< 0.0001 (****)
Median age, years (IQR)	1.04 (0.68–2.46)	12.88 (3.87-21)	n.a.	n.a.	< 0.0001 (****)
Not specified RNU4ATAC-related disorder, no. (%)	1 (2)	8 (16.67)	0.1	0.01–0.74	0.0305 (*)
RS, no. (%)	0 (0)	29 (60.42)	0	0-0.05	< 0.0001 (****)
LWS, no. (%)	0 (0)	5 (10.42)	0	0-0.63	0.0596 (ns)
MOPD1/TALS, no. (%)	49 (98)	6 (12.5)	343	47.4–3516	< 0.0001 (****)
Clinical and/or immunological evidence of immunodeficiency, no. (%)	8 (16)	36 (75)	0.06	0.02–0.18	< 0.0001 (****)
Hypo-/dysgammaglobulinemia, no. (%)	1 (2)	27 (56.25)	0.02	0-0.1	< 0.0001 (****)
Low B cells, no. (%)	0 (0)	22 (45.83)	0	0-0.1	< 0.0001 (****)
Severe/recurrent infections, no. (%)	7 (14)	32 (66.67)	0.08	0.03–0.23	< 0.0001 (****)
URTI, no. (%)	1 (2)	23 (47.92)	0.02	0-0.14	< 0.0001 (****)
LRTI, no. (%)	2 (4)	20 (41.67)	0.06	0.01–0.24	< 0.0001 (****)
GII, no. (%)	3 (6)	0 (0)	+inf.	0.85-+inf.	0.2555 (ns)
Encephalitis, no. (%)	4 (8)	0 (0)	+inf.	0.96-+inf.	0.1362 (ns)
Meningitis, no. (%)	0 (0)	1 (2.08)	0	0-8.64	0.9836 (ns)
Viral infections, no. (%)	2 (4)	10 (20.83)	0.16	0.03–0.65	0.0255 (*)
Fungal infections, no. (%)	0 (0)	3 (6.25)	0	0-1.09	0.2267 (ns)
Prenatal growth delay, no. (%)	50 (100)	37 (77.08)	+inf.	4.09-+inf.	0.0011 (**)
Postnatal growth delay, no. (%)	49 (98)	24 (50)	49	7.56–515.2	< 0.0001 (****)
Cranial dysmorphism, no. (%)	50 (100)	32 (66.7)	+inf.	5.99-+inf.	< 0.0001 (****)
Microcephaly, no. (%)	49 (98)	31 (64.58)	26.87	4-287	< 0.0001 (****)
Facial dysmorphism, no. (%)	33 (66)	1 (2.08)	91.24	13.84–949.1	< 0.0001 (****)
Limb dysmorphism, no. (%)	12 (24)	10 (20.83)	1.2	0.46–3.02	0.8939 (ns)
Digit dysmorphism, no. (%)	17 (34)	33 (68.75)	0.23	0.11–0.56	0.0012 (**)
Brachydactyly, no. (%)	9 (18)	27 (56.25)	0.17	0.07–0.42	0.0002 (***)
Clinodactyly, no. (%)	2 (4)	14 (29.17)	0.1	0.02–0.44	0.002 (**)
Syndactyly, no. (%)	2 (4)	3 (6.25)	0.63	0.11–3.19	0.9626 (ns)
Retinal dystrophy, no. (%)	4 (8)	20 (41.67)	0.12	0.04–0.38	0.0003 (***)
Strabismus, no. (%)	0 (0)	6 (12.5)	0	0-0.66	0.0309 (*)
Cataract, no. (%)	1 (2)	3 (6.25)	0.31	0.02–2.14	0.5807 (ns)
Optic nerve atrophy, no. (%)	0 (0)	2 (4.17)	0	0-2.06	0.457 (ns)
Brain malformation, no. (%)	37 (74)	16 (33.33)	5.69	2.27–13.34	0.0001 (****)
Gyration abnormalities, no. (%)	19 (38)	3 (6.25)	9.19	2.52–30.77	0.0004 (***)
Dysgenesis of corpus callosum, no. (%)	26 (52)	8 (16.67)	5.42	2.13–13.84	0.0005 (***)
Cerebellar hypoplasia, no. (%)	9 (18)	7 (14.58)	1.29	0.44–3.46	0.8539 (ns)
Vermian hypoplasia, no. (%)	5 (10)	4 (8.33)	1.22	0.33–4.19	0.9488 (ns)
Intracranial cysts, no. (%)	8 (16)	3 (6.25)	2.86	0.78–10.39	0.2269 (ns)
Sensineuronal hearing loss, no. (%)	1 (2)	6 (12.5)	0.14	0.01–0.96	0.1041 (ns)
Skeletal abnormality/ies, no. (%)	43 (86)	38 (79.17)	1.62	0.59–4.24	0.5312 (ns)
Epiphyseal-/spondyloepiphyseal dysplasia, no. (%)	3 (6)	29 (60.42)	0.04	0.01–0.15	0.0001 (****)
Joint contracture/dislocation, no. (%)	16 (32)	4 (8.33)	5.18	1.58–15.07	0.0079 (**)
Scoliosis/kyphosis, no. (%)	3 (6)	8 (16.67)	0.32	0.09–1.17	0.1763 (ns)
Agenesis of 12th ribs, no. (%)	2 (4)	3 (6.25)	0.63	0.11–3.19	0.9626 (ns)
Skin abnormalities, no. (%)	27 (54)	23 (47.92)	1.28	0.56–2.74	0.6891 (ns)
Dry skin, no. (%)	17 (34)	13 (27.08)	1.39	0.58–3.13	0.6007 (ns)
Eczema, no. (%)	1 (2)	10 (20.83)	0.08	0.01–0.51	0.0085 (**)
Chilblain-like, no. (%)	1 (2)	2 (4.17)	0.47	0.03–4.17	0.9714 (ns)
Hypoplastic/dysplastic nails, no. (%)	6 (12)	2 (4.17)	3.14	0.73–15.77	0.2952 (ns)
Cardiac septal defect, no. (%)	9 (18)	10 (20.83)	0.83	0.32–2.12	0.9211 (ns)
Endocrinological features, no. (%)	1 (2)	16 (33.33)	0.04	0-0.28	0.0001 (***)
Microdontia or enamel defects, no. (%)	5 (10)	6 (12.5)	0.78	0.24–2.98	0.9427 (ns)
Autoimmune/autoinflammatory features, no. (%)	0 (0)	7 (14.58)	0	0-0.5	0.016 (*)
Hepato-/splenomegaly, no. (%)	0 (0)	7 (14.58)	0	0-0.5	0.016 (*)
Hepatopathy, no. (%)	0 (0)	5 (10.42)	0	0-0.63	0.0596
Renal disease, no. (%)	2 (4)	3 (6.25)	0.63	0.11–3.19	0.9626

CI, confidence interval; GII, gastrointestinal infections; inf., infinity; LRTI, lower respiratory tract infections; LWS, Lowry-Wood syndrome; MOPD1, microcephalic osteodysplastic primordial dwarfism type 1; N, total number; n.a., not applicable; n.s. not significant; no., number; OR, odds ratio; RS, Roifman syndrome; TALS, Taybi-Linder syndrome; URTI, upper respiratory tract infections

^1^*p* < 0.05 *; *p* < 0.01 **; *p* < 0.001 ***; *p* < 0.0001 ****; ns, non-significant

## Discussion

Here we report four new cases with *RNU4ATAC*-related disease, including three adult patients, two of whom displayed a relatively late disease onset. The genetic diagnosis in these latter two patients was particularly unexpected given the absence of profound dysmorphic features, skeletal anomalies or neurodevelopmental delay. This observation suggests that, despite its classification as an IEI with dysmorphic features [47], genetic testing for *RNU4ATAC* variants should also be considered in patients with immunodeficiency, even in the absence of dysmorphic or skeletal features that would be typical for MOPD1 or RS. Nephropathy and hepatopathy, which we observed in three patients, have only rarely been described in individuals with *RNU4ATAC*-related disease. To our knowledge, persistent systemic inflammation, which was present in all four cases reported here, has not been yet described. Together with the unusually late disease onset in two of our patients, these findings broaden the phenotypic spectrum of *RNU4ATAC*-related disease.

One of the patients described here (P3) exhibited a combination of features, including immunodeficiency, renal disease and skeletal dysplasia that led to the clinical diagnosis of immuno-osseous dysplasia. However, WES failed to identify the causative variants since it focuses on protein-coding sequences. The diagnosis was ultimately achieved through WGS, highlighting its additive diagnostic value for IEIs and syndromic disorders involving non-coding RNA genes. Routine WGS and consideration of *RNU4ATAC* even in adult patients without typical features of MOPD1 or RS, would be expected to increase the number of patients with *RNU4ATAC*-related spliceosomopathies, enabling a more objective evaluation of the phenotypic spectrum of *RNU4ATAC*-related disease.

**Fig. 6:**
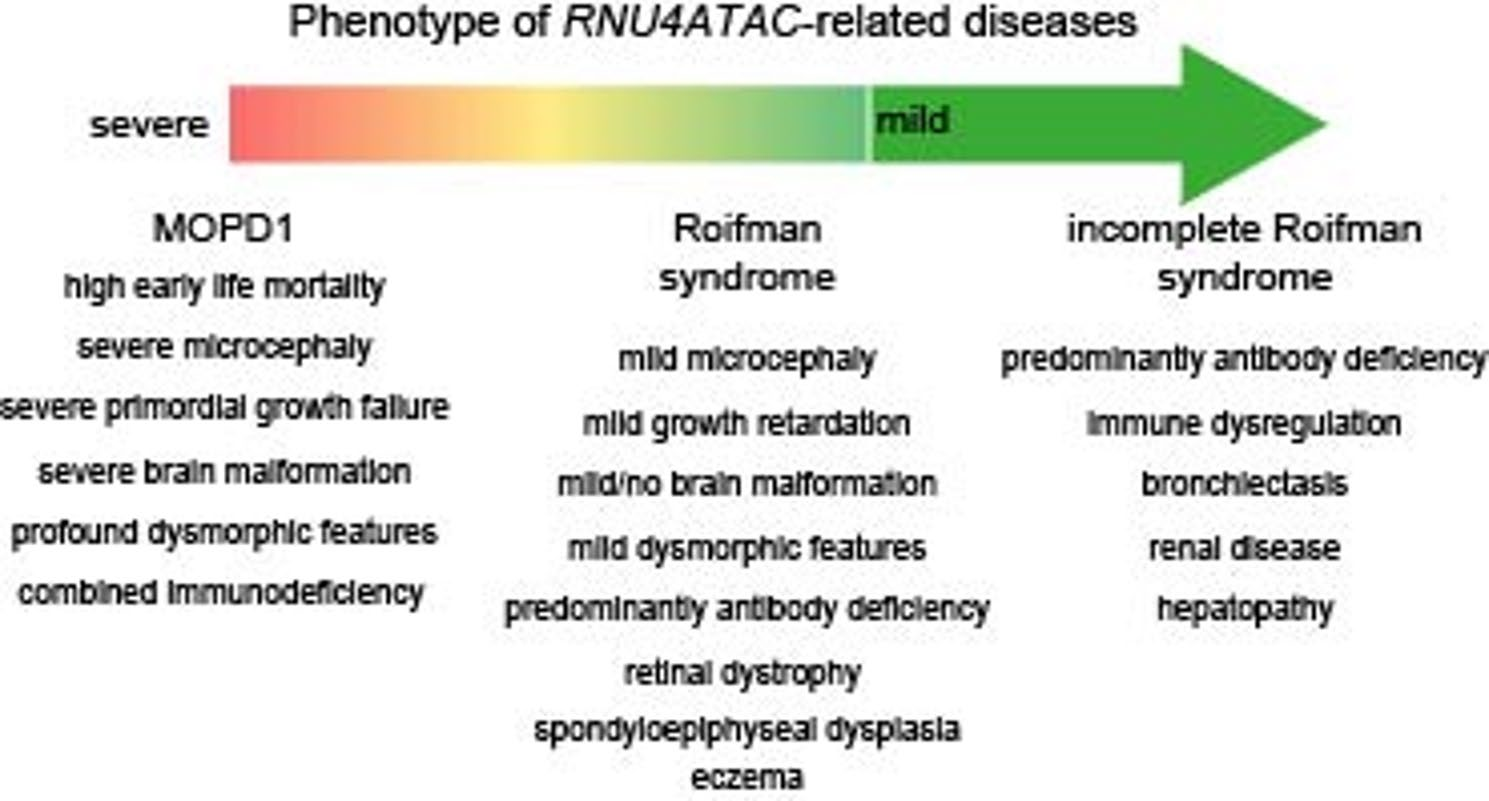
Perception of *RNU4ATAC*-related spliceosomopathies as a phenotypic continuum that should be expanded to encompass milder presentations and later-onset features

The re-evaluation of all published cases with *RNU4ATAC*-opathies confirmed a strong genotype-phenotype correlation: homozygous or compound heterozygous variants within the 5′ stem-loop region are associated with severe disease characterized by extreme dysmorphic features, primordial dwarfism, brain anomalies, and early mortality (i.e., MOPD1). In contrast, patients with at least one variant outside the 5′ stem–loop typically present with milder disease, including variable growth retardation, developmental delay, spondyloepiphyseal dysplasia, retinal dystrophy, and immunodeficiency – features more characteristic of RS. Nevertheless, some patients diagnosed with MOPD1 have been found to carry only one variant in the critical 5′ stem-loop region. In most of these cases, the variant outside the 5′ stem-loop was a deletion or duplication, expected to affect the 5′ stem-loop as well. The aforementioned genotype-phenotype correlation along with the distinct severity and adverse outcome of patients with variants exclusively within the 5’ stem-loop, underscore the clinical relevance of specific syndromes such as MOPD1 or RS over a general molecular term for all forms of *RNU4ATAC*-related disease. Yet, milder or infection-only cases (such as P2) with a rather incomplete RS, suggest the need for defining novel *RNU4ATAC*-related syndromes (Fig. 6).

The relative absence of reported immunodeficiency or retinal dystrophy in patients with variants exclusively located at the 5′ stem-loop, who were diagnosed with MOPD1, likely does not reflect an alternative pathophysiology, but rather a bias toward reporting the more prominent or congenital features, such as the dysmorphic features. Further, the early mortality associated with MOPD1 may preclude the onset of clinically evident immunodeficiency or other features with a relatively delayed onset, such as autoimmunity, retinopathy, renal disease or bronchiectasis.

The here presented cases suggest CKD as a relatively common feature of *RNU4ATAC*-opathies. Further, renal histopathological findings related to deleterious *RNU4ATAC* variants have been reported previously. Among previously reported cases, specific renal manifestations included congenital anomalies of the kidney and urinary tract (CAKUT) such as cystic or dysplastic kidneys, renal tubular dysfunction, specifically renal tubular acidosis and infection-associated chronic kidney disease, particularly renal atrophy, potentially due to recurrent infections or chronic kidney disease [39, 48]. All three patients presented with proteinuria, whereas none exhibited renal tubular acidosis. The differential diagnosis of proteinuria in this context is broad. In P1, it included renal involvement of ANCA-associated vasculitis. In case of P3, renal histopathology did not correspond to any known glomerulopathy and did not meet the criteria for podocytopathy. Further characterization of the renal phenotype is required to better understand the mechanisms causing CKD in these patients and determine whether they follow a consistent pattern.

Although immunodeficiency is less well characterized in severe *RNU4ATAC*-related phenotypes (MOPD1 or LWS), reported infectious complications in MOPD1, including severe viral infections with fatal outcomes, suggest a profound immunodeficiency. This contrasts with most RS cases or those with at least one deleterious *RNU4ATAC* variant outside the 5′ stem-loop region, where reported immunological findings and infections are rather consistent with antibody deficiency. Commonly reported immunological abnormalities in RS include hypogammaglobulinemia or dysgammaglobulinemia and the loss of antibody responses to vaccinations. The latter findings commonly associated with reduced total B cell counts and expanded transitional B-cell populations, a finding replicated in all four patients reported here. However, severe infectious complications leading to bronchiectasis (P2 and P3) or the presence of confluent warts (P2) are rather suggestive of a broader immunodeficiency.

Persistent systemic inflammation was reported in all four patients. The latter, together with the finding of increased PD-1 expression on T cells is consistent with T cell exhaustion, which may contribute to both cellular and humoral immunodeficiency in *RNU4ATAC*-related disease. The lack of clinical evidence for infection at testing suggests inflammation and immune exhaustion in the context of a disease-intrinsic immune dysregulation that drives T cell hyperactivation. The finding of elevated HLA-DR expression on T cells in all tested patients would be also consistent with chronic activation.

The broad clinical spectrum of *RNU4ATAC*-related disorders highlights the need to screen for diverse manifestations, including CNS abnormalities, neurodevelopmental delay and cardiological, dermatological as well as nephrological complications, at diagnosis and during follow-up. Accordingly, patient management requires interdisciplinary care involving multiple specialties. As demonstrated by the cases presented here, nephrological, pulmonary or rheumatological manifestations may be the initial or predominant features. Consequently, raising physician awareness of *RNU4ATAC*-related disorders is essential for improving diagnosis. Beyond their rarity and low awareness among clinicians, the absence or subtlety of dysmorphic or skeletal features and the requirement for WGS to detect non-coding variants, represent additional obstacles to diagnosing *RNU4ATAC*-related disorders.

## Supplementary Information

Below is the link to the electronic supplementary material.

Supplementary Material 1

## Data Availability

Data are available for formal research purposes only upon request to the corresponding author.

## References

[1] Turunen JJ, Niemelä EH, Verma B, Frilander MJ. The significant other: splicing by the minor spliceosome. Wiley Interdiscip Rev RNA. 2013;4:61–76. 23074130 10.1002/wrna.1141 PMC3584512

[2] Padgett RA. New connections between splicing and human disease. Trends Genet. 2012;28:147–54. 22397991 10.1016/j.tig.2012.01.001 PMC3319163

[3] Norppa AJ, Shcherbii MV, Frilander MJ. Connecting genotype and phenotype in minor spliceosome diseases. RNA. 2025;31:284–99. 39761998 10.1261/rna.080337.124 PMC11874965

[4] Shukla GC, Cole AJ, Dietrich RC, Padgett RA. Domains of human U4atac snRNA required for U12-dependent splicing in vivo. Nucleic Acids Res. 2002;30:4650–7. 12409455 10.1093/nar/gkf609 PMC135832

[5] Padgett RA, Shukla GC. A revised model for U4atac/U6atac snRNA base pairing. RNA. 2002;8:125–8. 11911359 10.1017/s1355838202017156 PMC1370236

[6] Liu S, Ghalei H, Lührmann R, Wahl MC. Structural basis for the dual U4 and U4atac snRNA-binding specificity of spliceosomal protein hPrp31. RNA. 2011;17:1655–63. 21784869 10.1261/rna.2690611 PMC3162331

[7] He H, Liyanarachchi S, Akagi K, Nagy R, Li J, Dietrich RC, et al. Mutations in U4atac snRNA, a component of the minor spliceosome, in the developmental disorder MOPD I. Science. 2011;332:238–40. 21474760 10.1126/science.1200587 PMC3380448

[8] Edery P, Marcaillou C, Sahbatou M, Labalme A, Chastang J, Touraine R, et al. Association of TALS developmental disorder with defect in minor splicing component U4atac snRNA. Science. 2011;332:240–3. 21474761 10.1126/science.1202205

[9] Merico D, Roifman M, Braunschweig U, Yuen RK, Alexandrova R, Bates A, et al. Compound heterozygous mutations in the noncoding RNU4ATAC cause Roifman Syndrome by disrupting minor intron splicing. Nat Commun. 2015;6:8718. 26522830 10.1038/ncomms9718 PMC4667643

[10] Farach LS, Little ME, Duker AL, Logan CV, Jackson A, Hecht JT, Bober M. The expanding phenotype of RNU4ATAC pathogenic variants to Lowry Wood syndrome. Am J Med Genet A. 2018;176:465–9. 29265708 10.1002/ajmg.a.38581 PMC6774248

[11] Duker A, Velasco D, Robertson N, Jackson A, DeFelice M, Bober MB. RNU4atac-opathy. GeneReviews^®^ [Internet]. 2023. 36795902

[12] Benoit-Pilven C, Besson A, Putoux A, Benetollo C, Saccaro C, Guguin J, et al. Clinical interpretation of variants identified in RNU4ATAC, a non-coding spliceosomal gene. PLoS ONE. 2020;15:e0235655. 32628740 10.1371/journal.pone.0235655 PMC7337319

[13] Heremans J, Garcia-Perez JE, Turro E, Schlenner SM, Casteels I, Collin R, et al. Abnormal differentiation of B cells and megakaryocytes in patients with Roifman syndrome. J Allergy Clin Immunol. 2018;142:630–46. 29391254 10.1016/j.jaci.2017.11.061

[14] Cologne A, Benoit-Pilven C, Besson A, Putoux A, Campan-Fournier A, Bober MB, et al. New insights into minor splicing-a transcriptomic analysis of cells derived from TALS patients. RNA. 2019;25:1130–49. 31175170 10.1261/rna.071423.119 PMC6800510

[15] Olthof AM, White AK, Mieruszynski S, Doggett K, Lee MF, Chakroun A, et al. Disruption of exon-bridging interactions between the minor and major spliceosomes results in alternative splicing around minor introns. Nucleic Acids Res. 2021;49:3524–45. 33660780 10.1093/nar/gkab118 PMC8034651

[16] Tabib A, Richmond CM, McGaughran J. Delineating the phenotype of RNU4ATAC-related spliceosomopathy. Am J Med Genet A. 2023;191:1094–100. 36622817 10.1002/ajmg.a.63110

[17] Khatri D, Putoux A, Cologne A, Kaltenbach S, Besson A, Bertiaux E, et al. Deficiency of the minor spliceosome component U4atac snRNA secondarily results in ciliary defects in human and zebrafish. Proc Natl Acad Sci U S A. 2023;120:e2102569120. 36802443 10.1073/pnas.2102569120 PMC9992838

[18] Dogru D, Dogru Y, Atschekzei F, Elsayed A, Dubrowinskaja N, Ernst D, et al. Reappraisal of IgG subclass deficiencies: a retrospective comparative cohort study. Front Immunol. 2025;16:1552513. 10.3389/fimmu.2025.1552513. 40313941 10.3389/fimmu.2025.1552513 PMC12043879

[19] Elsayed A, von Hardenberg S, Atschekzei F, Siek P, Witte T, Sogkas G, Ringshausen FC. A novel hemizygous nonsense variant in DOCK11 causes systemic inflammation and immunodeficiency. Clin Immunol. 2025;276:110504. 10.1016/j.clim.2025.110504. 40274249 10.1016/j.clim.2025.110504

[20] Richards S, Aziz N, Bale S, Bick D, Das S, Gastier-Foster J, et al. Standards and guidelines for the interpretation of sequence variants: a joint consensus recommendation of the American College of Medical Genetics and Genomics and the Association for Molecular Pathology. Genet Med. 2015;17:405–24. 25741868 10.1038/gim.2015.30 PMC4544753

[21] Page MJ, McKenzie JE, Bossuyt PM, Boutron I, Hoffmann TC, Mulrow CD, et al. The PRISMA 2020 statement: an updated guideline for reporting systematic reviews. BMJ. 2021;372:n71. 10.1136/bmj.n71. 33782057 10.1136/bmj.n71 PMC8005924

[22] Elhassan EAE, Cormican S, Osman SM, Sarihan S, Teltsh O, Poynton FE, Griffin MD, Casserly L, McCann E, Bleyer AJ, Sr, Kmoch S, Živná M, Benson KA, Cavalleri GL, Conlon PJ. Characterization of Monogenic Kidney Disease in Older Patients With CKD. Kidney Int Rep. 2025;10:2140–52. 40677324 10.1016/j.ekir.2025.04.017 PMC12266177

[23] Elston L, Fegan C, Hills R, Hashimdeen SS, Walsby E, Henley P, et al. Increased frequency of CD4 + PD-1 + HLA-DR + T cells is associated with disease progression in CLL. Br J Haematol. 2020;188:872–80. 31702049 10.1111/bjh.16260

[24] Gauthier LW, Gossez M, Malcus C, Viel S, Monneret G, Bordonné R, et al. B-cell immune deficiency in twin sisters expands the phenotype of MOPDI. Clin Genet. 2024;106:476–82. 38837402 10.1111/cge.14571

[25] Poli A, Michel T, Thérésine M, Andrès E, Hentges F, Zimmer J. CD56bright natural killer (NK) cells: an important NK cell subset. Immunology. 2009;126:458–65. 19278419 10.1111/j.1365-2567.2008.03027.x PMC2673358

[26] Robertson N, Joshi A, Ritchie F, van der Schim I, Royan D, Duker AL, et al. Mutations in RNU4ATAC Are Associated With Chilblain-Like Lesions and Enhanced Type I Interferon Signalling. Eur J Immunol. 2025;55:e202451518. 40415209 10.1002/eji.202451518 PMC12104551

[27] Hallermayr A, Graf J, Koehler U, Laner A, Schönfeld B, Benet-Pagès A, Holinski-Feder E. Extending the critical regions for mutations in the non-coding gene RNU4ATAC in another patient with Roifman Syndrome. Clin Case Rep. 2018;6:2224–8. 30455926 10.1002/ccr3.1830 PMC6230649

[28] Dinur Schejter Y, Ovadia A, Alexandrova R, Thiruvahindrapuram B, Pereira SL, Manson DE, et al. A homozygous mutation in the stem II domain of *RNU4ATAC* causes typical Roifman syndrome. NPJ Genom Med. 2017;2:23. 29263834 10.1038/s41525-017-0024-5 PMC5677950

[29] Xi Q, Plaza Enriquez LJ, Tanni NU, Patsias I. Underdiagnosed Roifman syndrome manifested as non-ischaemic cardiomyopathy: a case report. ESC Heart Fail. 2023;10:3195–8. 37666272 10.1002/ehf2.14518 PMC10567627

[30] Almentina Ramos Shidi F, Cologne A, Delous M, Besson A, Putoux A, et al. Mutations in the non-coding RNU4ATAC gene affect the homeostasis and function of the Integrator complex. Nucleic Acids Res. 2023;51:712–27. 36537210 10.1093/nar/gkac1182 PMC9881141

[31] Ballios BG, Mandola A, Tayyib A, Tumber A, Garkaby J, Vong L, et al. Deep phenotypic characterization of the retinal dystrophy in patients with RNU4ATAC-associated Roifman syndrome. Eye (Lond). 2023;37:3734–42. 37225827 10.1038/s41433-023-02581-1 PMC10697969

[32] Clifford D, Moloney F, Leahy TR, Murray DM. Roifman syndrome: a description of further immunological and radiological features. BMJ Case Rep. 2022;15:e249109. 35450878 10.1136/bcr-2022-249109 PMC9024203

[33] McMillan HJ, Davila J, Osmond M, Chakraborty P, Care4Rare Canada Consortium, Boycott KM, et al. Whole genome sequencing identifies pathogenic RNU4ATAC variants in a child with recurrent encephalitis, microcephaly, and normal stature. Am J Med Genet A. 2021;185:3502–6. 34405953 10.1002/ajmg.a.62457

[34] Shelihan I, Ehresmann S, Magnani C, Forzano F, Baldo C, Brunetti-Pierri N, Campeau PM. Lowry-Wood syndrome: further evidence of association with RNU4ATAC, and correlation between genotype and phenotype. Hum Genet. 2018;137:905–9. 30368667 10.1007/s00439-018-1950-8

[35] Dinur Schejter Y, Merico D, Manson D, Reid B, Vong L. A novel mutation in Roifman syndrome redefines the boundaries of the Sm protein-binding site. LymphoSign J. 2016;3:159–63.

[36] Hagiwara H, Matsumoto H, Uematsu K, Zaha K, Sekinaka Y, Miyake N, et al. Immunodeficiency in a patient with microcephalic osteodysplastic primordial dwarfism type I as compared to Roifman syndrome. Brain Dev. 2021;43:337–42. 33059947 10.1016/j.braindev.2020.09.007

[37] Bogaert DJ, Dullaers M, Kuehn HS, Leroy BP, Niemela JE, De Wilde H, et al. Early-onset primary antibody deficiency resembling common variable immunodeficiency challenges the diagnosis of Wiedeman-Steiner and Roifman syndromes. Sci Rep. 2017;7:3702. 28623346 10.1038/s41598-017-02434-4 PMC5473876

[38] Krøigård AB, Jackson AP, Bicknell LS, Baple E, Brusgaard K, Hansen LK, Ousager LB. Two novel mutations in RNU4ATAC in two siblings with an atypical mild phenotype of microcephalic osteodysplastic primordial dwarfism type 1. Clin Dysmorphol. 2016;25:68–72. 26641461 10.1097/MCD.0000000000000110 PMC4772811

[39] Krøigård AB, Frost M, Larsen MJ, Ousager LB, Frederiksen AL. Bone structure in two adult subjects with impaired minor spliceosome function resulting from RNU4ATAC mutations causing microcephalic osteodysplastic primordial dwarfism type 1 (MOPD1). Bone. 2016;92:145–9. 27591150 10.1016/j.bone.2016.08.023

[40] Ferrell S, Johnson A, Pearson W. Microcephalic osteodysplastic primordial dwarfism type 1. BMJ Case Rep. 2016;2016:bcr2016215502. 10.1136/bcr-2016-215502. 27312855 10.1136/bcr-2016-215502 PMC4932407

[41] Putoux A, Alqahtani A, Pinson L, Paulussen AD, Michel J, Besson A, et al. Refining the phenotypical and mutational spectrum of Taybi-Linder syndrome. Clin Genet. 2016;90:550–5. 27040866 10.1111/cge.12781

[42] Abdel-Salam GM, Emam BA, Khalil YM, Abdel-Hamid MS. Long-term survival in microcephalic osteodysplastic primordial dwarfism type I: Evaluation of an 18-year-old male with g.55G > A homozygous mutation in RNU4ATAC. Am J Med Genet A. 2016;170A:277–82. 26419500 10.1002/ajmg.a.37409

[43] Kilic E, Yigit G, Utine GE, Wollnik B, Mihci E, Nur BG, Boduroglu K. A novel mutation in RNU4ATAC in a patient with microcephalic osteodysplastic primordial dwarfism type I. Am J Med Genet A. 2015;167A:919–21. 25735804 10.1002/ajmg.a.36955

[44] Abdel-Salam GM, Abdel-Hamid MS, Hassan NA, Issa MY, Effat L, Ismail S, et al. Further delineation of the clinical spectrum in RNU4ATAC related microcephalic osteodysplastic primordial dwarfism type I. Am J Med Genet A. 2013;161A:1875–81. 23794361 10.1002/ajmg.a.36009

[45] Nagy R, Wang H, Albrecht B, Wieczorek D, Gillessen-Kaesbach G, Haan E, et al. Microcephalic osteodysplastic primordial dwarfism type I with biallelic mutations in the RNU4ATAC gene. Clin Genet. 2012;82:140–6. 21815888 10.1111/j.1399-0004.2011.01756.x PMC3816635

[46] Abdel-Salam GM, Miyake N, Eid MM, Abdel-Hamid MS, Hassan NA, Eid OM, et al. A homozygous mutation in RNU4ATAC as a cause of microcephalic osteodysplastic primordial dwarfism type I (MOPD I) with associated pigmentary disorder. Am J Med Genet A. 2011;155A:2885–96. 21990275 10.1002/ajmg.a.34299

[47] Poli MC, Aksentijevich I, Bousfiha AA, Cunningham-Rundles C, Hambleton S, Klein C, et al. Human inborn errors of immunity: 2024 update on the classification from the International Union of Immunological Societies Expert Committee. J Hum Immun. 2025;1:e20250003. 10.70962/jhi.20250003. 41608114 10.70962/jhi.20250003 PMC12829761

[48] Eason J, Hall CM, Trounce JQ. Renal tubular leakage complicating microcephalic osteodysplastic primordial dwarfism. J Med Genet. 1995;32:234–5. 7783178 10.1136/jmg.32.3.234 PMC1050326

