# Supplementary material for: Phenotypic spectrum of RNU4ATAC-related spliceosomopathies: four novel cases and integrated reevaluation of previously reported patients
Source: Orphanet J Rare Dis. 2026 Mar 10;21:137. doi: 10.1186/s13023-026-04300-x (PMC13063612; doi:10.1186/s13023-026-04300-x)
Supplement: Supplementary file 1 — Supplementary Material 1 [file 13023_2026_4300_MOESM1_ESM.docx]

**Supplemental Material**

**
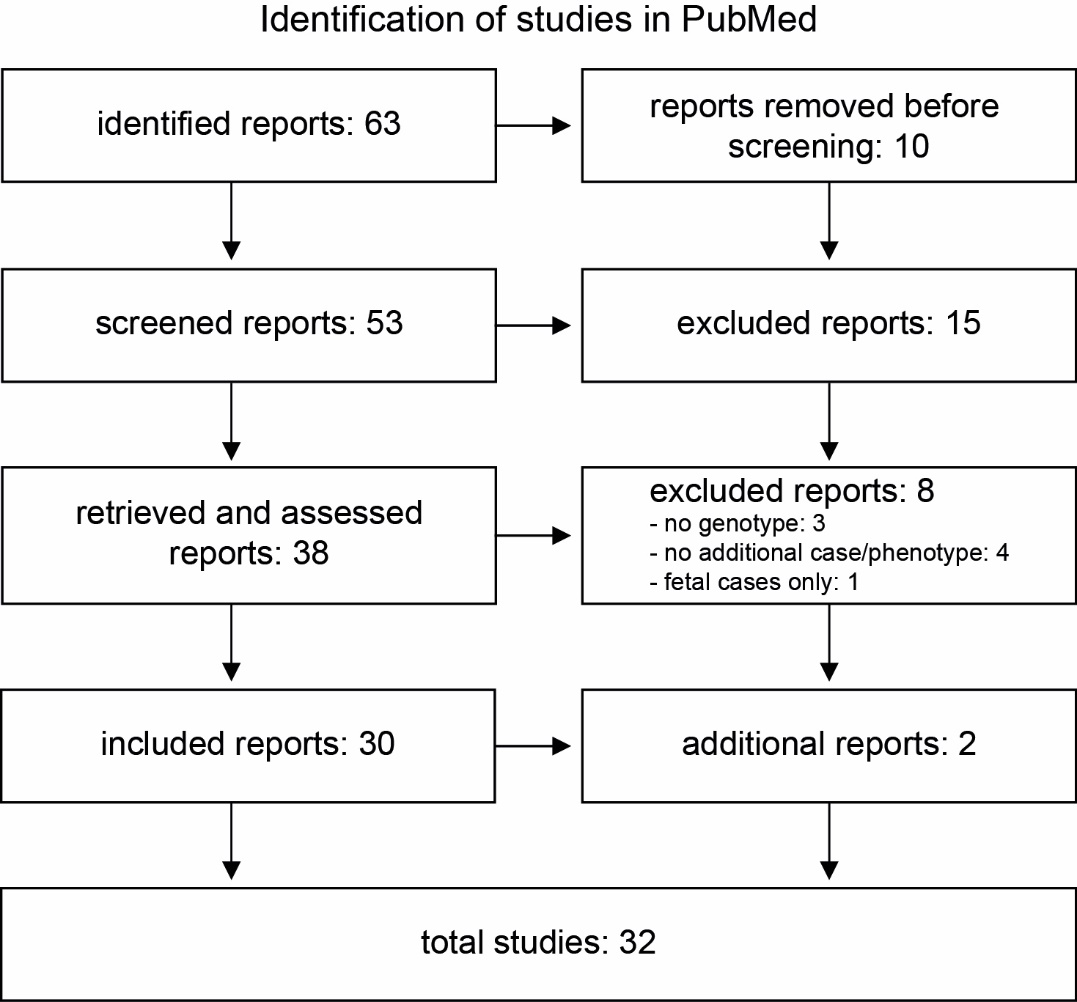
**

**Suppl. Figure 1:** PRISMA flow diagram on literature review

**Suppl. Table 1**: Overview of demographic data, clinical diagnosis and genotype in 98 patients with RNU4ATAC-related spliceosomopathies

| **Patient ID** | **Age (years)** | **Sex /**  **Kinship** | **Origin** | ***RNU4ATAC*-related disorder** | ***RNU4ATAC* variant** | **References** |
| --- | --- | --- | --- | --- | --- | --- |
| 1 | 3 | f | Pakistani | n.a. | n.40C>T; n.65C>T | 26 |
| 2 | 12 | m | n.a. | n.a. | n.13_15del; n.13C>T | 26 |
| 3 | 2 | f | n.a. | MOPD1 | n.108_126del; n.111G>A | 23 |
| 4 | 2 | f (twin sister of 3) | n.a. | MOPD1 | n.108_126del; n.111G>A | 23 |
| 5 | 20 | m | n.a. | RS | n.47T>G; n.118T>C | 29 |
| 6 | n.a. | f (sister of 5) | n.a. | RS | n.47T>G; n.118T>C | 29 |
| 7 | 2 | m | n.a. | RS | n.17G>A; n.116A>G | 30 |
| 8 | 8 | f | n.a. | RS | n.16G>A; n.16G>A | 30,31 |
| 9 | 13 | m | n.a. | RS | n. 13C>T; n.48G>A | 30 |
| 10 | 13 | m | Tamilar | RS | n.17G>A; n.116A>G | 30 |
| 11 | 25 | m | n.a. | RS | n. 13C>T; n.29T>C | 30 |
| 12 | 20 | f | n.a. | RS | n. 13C>T; n.46G>A | 30 |
| 13 | 26 | m | Italian | RS | n. 13C>T; n.48G>A | 9, 30 |
| 14 | 26 | m | n.a. | RS | n. 16G>A; n.50 G>A; n.55G>A | 30 |
| 15 | 33 | m | English | RS | n.13C>T; n.37G>A | 9, 30 |
| 16 | 41 | m (brother of 15) | English | RS | n.13C>T; n.37G>A | 9, 30 |
| 17 | 3 | m | Algerian | n.a. | n.16G>A; n.16G>A | 17 |
| 18 | 0 | f | Turkish | n.a. | n.16G>A; n.16G>A | 17 |
| 19 | 4 | f | n.a. | n.a. | n.16G>A; n.51G>A | 17 |
| 20 | 17 | m | Turkish | n.a. | n.16G>A; n.33C>G | 17 |
| 21 | 10 | f | Turkish | n.a. | n.16G>A; n.33C>G | 17 |
| 22 | n.a. | f | Australian-Caucasian | n.a. | n. 6T>C; n.48G>A | 16 |
| 23 | 9 | m | n.a. | RS | n.13C>T; n.48G>A | 28 |
| 24 | 12 | m (brother of 23) | n.a. | RS | n.13C>T; n.48G>A | 28 |
| 25 | 10 | f | central African | n.a. | n.55G>A; n.55G>A | 32 |
| 26 | 1.5 | m | n.a. | RS | n.17G>A; n.116A>G | 15 |
| 27 | 28 | m | n.a. | LWS | n.114G>C; n.120T>G | 33 |
| 28 | 19 | m | n.a. | LWS | n.8C>A; n.53C>T | 33 |
| 29 | 12 | m | Tamilar | RS | n.17G>A; n.116A>G | 34 |
| 30 | 18 | f | Japanese | MOPD1 | n.50G>A; n.55G>A | 35 |
| 31 | 6 | f | n.a. | RS | n.13C>T; n.116A>T | 27 |
| 32 | 12 | m | Belgian European | RS | n.16G>A; n.46G>A | 13 |
| 33 | 13 | f (sister of 32) | Belgian European | RS | n.16G>A; n.46G>A | 13 |
| 34 | 38 | m | Belgian European | RS | n. 16G>A; n.16G>A | 13 |
| 35 | 3.5 | f | n.a. | LWS | n.5A>C; n.51G>A | 36 |
| 36 | 14 | m | n.a. | LWS | n. 46G>A; n.111G>A | 36 |
| 37 | 15 | f (sister of 36) | n.a. | LWS | n. 46G>A; n.111G>A | 36 |
| 38 | 17 | m | Belgian | RS | n.13C>T; n.116A>T | 10, 37 |
| 39 | 14 | f (sister of 38) | Belgian | RS | n.13C>T; n.116A>T | 10, 37 |
| 40 | 24 | f | Caucasian | MOPD1 | n.40C>T; n.16_100dup | 38 |
| 41 | 17 | m (brother of 50) | Caucasian | MOPD1 | n.40C>T; n.16_100dup | 38 |
| 42 | 2 | f | n.a. | MOPD1 | n. 51G>A; n.51G>A | 39 |
| 43 | 6 | n.a. | India | MOPD1 | n.51G>A; n.55G>A | 40 |
| 44 | 1.6 | n.a. | Malta | MOPD1 | n.51G>A; n.51G>A | 40 |
| 45 | 0.5 | n.a. | Turkish | MOPD1 | n.51G>A; n.51G>A | 40 |
| 46 | 6 | n.a. | Rwanda | MOPD1 | n.51G>A; n.51G>A | 40 |
| 47 | 3.5 | f | Lebanese-Australian | RS | n.16G>A; n.51G>A | 9 |
| 48 | 2.5 | m (brother of 47) | Lebanese-Australian | RS | n.16G>A; n.51G>A | 9 |
| 49 | 4 | m | Albanian | RS | n.8C>T; n.118T>C | 9 |
| 50 | 18 | m | n.a. | MOPD1 | n.55G>A; n.55G>A | 41 |
| 51 | 3 | m | n.a. | MOPD1 | n.46G>A; n.46G>A | 42 |
| 52 | 1.8 | m | Egyptian | MOPD1 | n.55G>A; n.55G>A | 43 |
| 53 | 2.3 | m (brother of 52) | Egyptian | MOPD1 | n.55G>A; n.55G>A | 43 |
| 54 | 1.5 | m | Egyptian | MOPD1 | n.55G>A; n.55G>A | 43 |
| 55 | 0.7 | f | Egyptian | MOPD1 | n.51G>C; n.51G>C | 43 |
| 56 | 1 | f (sister of 55) | Egyptian | MOPD1 | n.51G>C; n.51G>C | 43 |
| 57 | 1.7 | m | n.a. | MOPD1 | n.66G>C; n.124G>A | 43 |
| 58 | 1 | m | Yemeni | MOPD1 | n.55G>A; n.55G>A | 43 |
| 59 | 2.8 | f (sister of 58) | Yemeni | MOPD1 | n.55G>A; n.55G>A | 43 |
| 60 | 1.5 | f | Amish | MOPD1 | n.51G>A; n.51G>A | 44 |
| 61 | 0.3 | f | Amish | MOPD1 | n.51G>A; n.51G>A | 44 |
| 62 | 0.2 | f | Amish | MOPD1 | n.51G>A; n.51G>A | 44 |
| 63 | 0.8 | f | Amish | MOPD1 | n.51G>A; n.51G>A | 44 |
| 64 | 0.8 | m | Amish | MOPD1 | n.51G>A; n.51G>A | 44 |
| 65 | n.a | f | Amish | MOPD1 | n.51G>A; n.51G>A | 44 |
| 66 | n.a. | n.a. | Amish | MOPD1 | n.51G>A; n.51G>A | 44 |
| 67 | n.a. | n.a. | Amish | MOPD1 | n.51G>A; n.51G>A | 44 |
| 68 | 0.8 | m | Amish | MOPD1 | n.51G>A; n.51G>A | 44 |
| 69 | 0.8 | f | Amish | MOPD1 | n.51G>A; n.51G>A | 44 |
| 70 | 0.4 | f | Amish | MOPD1 | n.51G>A; n.51G>A | 44 |
| 71 | 0.7 | f | Amish | MOPD1 | n.51G>A; n.51G>A | 44 |
| 72 | 1 | f | Amish | MOPD1 | n.51G>A; n.51G>A | 44 |
| 73 | 0.7 | f | Amish | MOPD1 | n.51G>A; n.51G>A | 44 |
| 74 | 0.1 | m | Maltese | MOPD1 | n.51G>A; n.51G>A | 44, 45 |
| 75 | 9 | f | German | MOPD1 | n.55G>A; n.55G>A | 44, 45 |
| 76 | 12.8 | m | German | MOPD1 | n.30G>A; n.111G>A | 44, 45 |
| 77 | 5 | m | Egyptian | MOPD1 | n.55G>A; n.55G>A | 7 |
| 78 | 2.5 | m (brother of 77) | Egyptian | MOPD1 | n.55G>A; n.55G>A | 7 |
| 79 | 0.9 | m | Algerian | MOPD1 | n. 51G>A; n.51G>A | 46 |
| 80 | 0.8 | m (brother of 79) | Algerian | MOPD1 | n. 51G>A; n.51G>A | 46 |
| 81 | 1.2 | f | Turkish | MOPD1 | n. 51G>A; n.51G>A | 46 |
| 82 | 0.6 | f | Maroccan | MOPD1 | n. 51G>A; n.51G>A | 40 |
| 83 | 1.1 | f | Maroccan | MOPD1 | n. 51G>A; n.51G>A | 46 |
| 84 | 2.3 | f (sister of 83) | Maroccan | MOPD1 | n. 51G>A; n.51G>A | 46 |
| 85 | 0.4 | m | Indian | MOPD1 | n. 51G>A; n.51G>A | 46 |
| 86 | 0.5 | m | Caucasian from north America | MOPD1 | n. 50G>A; n.51G>A | 46 |
| 87 | 2.3 | f | Caucasian from north America | MOPD1 | n. 50G>C; n.51G>A | 46 |
| 88 | n.a. | f | Amish | MOPD1 | n.51G>A; n.51G>A | 45 |
| 89 | n.a. | f | Amish | MOPD1 | n.51G>A; n.51G>A | 45 |
| 90 | n.a. | f | Amish | MOPD1 | n.51G>A; n.51G>A | 45 |
| 91 | n.a. | f | Amish | MOPD1 | n.51G>A; n.51G>A | 45 |
| 92 | n.a. | f | Amish | MOPD1 | n.51G>A; n.51G>A | 45 |
| 93 | n.a. | f | Amish | MOPD1 | n.51G>A; n.51G>A | 45 |
| 94 | n.a. | m | Amish | MOPD1 | n.51G>A; n.51G>A | 45 |
| 95 | 43.2 | f | German | RS | n.8C>T; n.51G>A | this publication (P1) |
| 96 | 32.3 | f | German | RS | n.8C>T; n.37G>A | this publication (P2) |
| 97 | 37.6 | f | German | RS | n.13C>T; n.117A>G | this publication (P3) |
| 98 | 8 | m | German | RS | n.13C>T; n.51G>A | this publication  (P4) |
| F, female; ID, identification number; LWS, Lowry-Wood syndrome; M, male; MOPD1, microcephalic osteodysplastic primordial dwarfism type 1; n.a., not available; RS, Roifman syndrome | | | | | | |
